# Impact of type of anticoagulant on clinical outcomes in cancer patients who had atrial fibrillation

**DOI:** 10.1038/s41598-023-38071-3

**Published:** 2023-07-06

**Authors:** Chatree Chai-Adisaksopha, Alexandre H. Watanabe, Piyameth Dilokthornsakul, Leenhapong Navaravong, Daniel M. Witt, Nathorn Chaiyakunapruk

**Affiliations:** 1 Department of Internal Medicine, Chiang Mai University, Chiang Mai, Thailand; 2 Department of Pharmacotherapy, Pharmacotherapy Outcomes Research Center, University of Utah, Salt Lake City, UT USA; 3 Center for Medical and Health Technology Assessment (CM-HTA), Department of Pharmaceutical Care, Faculty of Pharmacy, Chiang Mai University, Chiang Mai, Thailand; 4 Division of Cardiovascular Medicine, University of Utah, Salt Lake City, USA; 5 IDEAS Center, Veterans Affairs Salt Lake City Healthcare System, Salt Lake City, UT USA

**Keywords:** Cancer, Cardiology, Health care, Medical research

## Abstract

To date, evidence on optimal anticoagulant options in patients with AF who concurrently have active cancer remains elusive. To describe anticoagulant patterns and clinical outcomes among patients with a concomitant diagnosis of AF and cancer. Data were obtained from the University of Utah and Huntsman Cancer Institute (HCI) Hospitals. Patients were included if they had diagnosis of AF and cancer. Outcome was type and pattern of anticoagulant. Clinical outcomes were stroke, bleeding and all-cause mortality. From October 1999 to December 2020, there were 566 AF patients who concurrently had active cancer. Mean age ± standard deviation was 76.2 ± 10.7 and 57.6% were males. Comparing to warfarin, patients who received direct oral anticoagulant (DOACs) were associated with similar risk of stroke (adjusted hazard ratio, aHR 0.8, 95% confidence interval [CI] 0.2–2.7, P = 0.67). On contrary, those who received low-molecular-weight heparin (LMWH) were associated with significantly higher risk of stroke comparing to warfarin (aHR 2.4, 95% CI 1.0–5.6, P = 0.04). Comparing to warfarin, DOACs and LMWH was associated with similar risk of overall bleeding with aHR 1.1 (95% CI 0.7–1.6, P = 0.73) and aHR 1.1 (95% CI 0.6–1.7, P = 0.83), respectively. Patients who received LMWH but not DOACs were associated with increased risk of death as compared to warfarin, aHR 4.5 (95% CI 2.8–7.2, P < 0.001) and 1.2 (95% CI 0.7–2.2, P = 0.47). In patients with active cancer and AF, LMWH, compared to warfarin, was associated with an increased risk of stroke and all-cause mortality. Furthermore, DOACs was associated with similar risk of stroke, bleeding and death as compared to warfarin.

## Introduction

Atrial fibrillation (AF) is the most common cardiac arrhythmia that affects more than 33 million people worldwide^1^. AF is associated with increased morbidity including cardioembolic stroke, systemic arterial embolism, myocardial infarction, heart failure, dementia, and increased risk of mortality^2–6^. Although AF affects adults at all ages, the incidence of AF is higher in older individuals. The prevalence of AF increased from 0.1% in younger adults (age < 55) to 9% in adults aged 80 years or older^7^. Anticoagulant therapy is the mainstay for the prevention of stroke and systemic embolism in patients with AF and additional stroke risk factors. Examples of anticoagulation therapy includes warfarin, low-molecular-weigh heparin (LMWH), unfractionated heparin (UFH), and direct oral anticoagulants (DOACs) such as dabigatran, apixaban, rivaroxaban, and edoxaban^8^.

Advanced age is also associated with an increased risk of developing cancer. Given that both AF and cancer are more prevalent in older adults and numerous older adults have multiple chronic conditions, the co-occurrence of AF and cancer can be expected. A recent nationwide population-based study demonstrated increased risk of incident AF with adjusted hazard ratio of 1.63 (95% confident interval [CI] 1.61–1.66) in patients with cancer^9^.

The use of oral anticoagulants in cancer patients is impeded by potential drug–drug interactions with cancer treatments, including chemotherapy and supportive medications such as antibiotics or antifungals. Furthermore, cancer patients often experience complications such as renal impairment and thrombocytopenia during cancer therapy, increasing their vulnerability to bleeding complications associated with anticoagulant treatment. Consequently, selecting the appropriate anticoagulant for this patient population requires careful consideration, recognizing the unique challenges posed by cancer. To date, evidence on optimal anticoagulant options in patients with AF who concurrently have active cancer remains elusive. Existing clinical guidelines on prevention VTE in AF patients do not provide recommendations for patients with AF and active cancer.

To address this information gap, we proposed to utilize real-world data to describe anticoagulation treatment patterns and identify the appropriate pharmacological treatment options based on drug interactions between anticoagulants, especially DOACs, and neoplastic agents and also based on the clinical outcomes associated with each anticoagulant treatment.

## Materials and methods

### Data source

Data used in this study were obtained from the University of Utah and Huntsman Cancer Institute (HCI) Hospitals which are major health centers serving the Intermountain West region. The University of Utah health system and HCI’s tumor registry provide a rich data source, the Electronic data warehouse (EDW), which is maintained by the University of Utah Health Science Data Resource Center. The EDW contains a broad range of clinical data from more than 2 million patients, such as patients’ demographics, clinical encounters, disease diagnoses, medical procedures and treatments, lab tests, list of medications, physicians’ notes, and health outcomes.

### Patients

Patients were included in the study if they were aged 18 years old and older, had a diagnosis of AF defined as having at least two diagnosis code of ICD-9 or ICD-10 encounters suggestive of atrial fibrillation (I48.x) within the study period, use of at least one anticoagulation therapy (i.e. warfarin, dabigatran, rivaroxaban, apixaban, edoxaban, enoxaparin, dalteparin, or tinzaparin), and had any neoplasm diagnosis defined as having at least two diagnosis code of ICD-9 or ICD-10 encounters suggestive of cancer (C00.x–D49.x) within the study period. Patients with valvular AF were not included. Cancer patients who did not receive anticoagulant at the date of cancer diagnosis were excluded from the study.

### Data collection

We collected the following data; patient demographics, type and primary site of cancer, date of cancer diagnosis, date of AF diagnosis, CHA_2_DS_2_-VASc score, serum creatinine and creatinine clearance, medical history included previous ischemic heart disease, stroke, peripheral arterial disease, diabetes, hypertension, dyslipidemia and chronic kidney disease. Medical history in individual patients was retrieved using ICD-9 or ICD-10 codes for any of these conditions in the 6 months prior to the cancer diagnosis. Index date was the date when patients were firstly diagnosed with cancer. We collected all anticoagulants prescribed in individual patients. The date of starting, switching, and of stopping anticoagulant was collected.

### Outcomes

The primary outcome of interest was type of anticoagulant prescription before and after the diagnosis of cancer in individual patients. We divided patients into 4 groups according to the pattern of anticoagulant prior to index date of cancer diagnosis; *group 1* patients who did not receive anticoagulant therapy (no anticoagulant group), *group 2* patients who received warfarin before cancer diagnosis (warfarin group), *group 3* patients who received DOACs before cancer diagnosis (DOAC group) and *group 4* patients who received either LMWH or UFH (LMWH/UFH group) before cancer diagnosis.

The primary clinical outcomes were the occurrence of stroke after index date of cancer diagnosis. The secondary clinical outcomes were bleeding (gastrointestinal bleeding, intracranial bleeding and other bleeding) that required hospitalization, and all-cause mortality. Stroke and bleeding events were identified using ICD-9 or ICD-10 codes which occurred following the index date.

### Statistical analysis

Descriptive statistics were used to summarize patient characteristics and treatment patterns in the cohort. Mean and standard deviation were used to report continuous variables. Frequency and percentages were used to report categorical variables.

Patients in the cohort were followed up until they developed a clinical outcome of interest or until the end of study period, whichever came first. Patients who did not develop a clinical outcome of interest or whose last encounter was identified by the end of the study period were considered censored.

Given that patients were likely to switch anticoagulants during the follow-up period, we conducted the primary analysis using marginal structural models (MSM). The MSM is a statistical technique that accounts for potential biases introduced by time-dependent confounders when analyzing the effect of time-varying exposures on outcomes^10,11^. In our cohort, patients experienced changes in clinical variables, such as serum creatinine, body weight, or hemoglobin, which could act as potential confounding variables. These changes were likely to affect the type of anticoagulant received. To implement the MSM, we assigned weights to each participant at each time point based on their anticoagulant exposure history. These weights were used to balance the influence of time-varying confounders, thus enabling a more accurate estimation of the treatment effect on the outcome. We then calculated inverse probability weights (IPWs) by taking the inverse of the estimated propensity score, representing the conditional probability of receiving a particular exposure given the participants' covariates. Subsequently, we performed a weighted outcome analysis, incorporating the individual-specific weights, using the Cox proportional hazard model to estimate the causal effect of the exposure on the outcome^10,11^. Hazard ratios (HRs) and 95% confidence intervals (CIs) were reported. The events were presented as events per 100 patient-years. We compared the risk of developing outcomes across all types of anticoagulants. The sensitivity analysis was performed by excluding participants who were enrolled before 2010 (DOAC was not yet approved for stroke and systemic embolism prevention in AF patients). All analyses were conducted using STATA version 15.1 (StataCorp. 2017. Stata Statistical Software: Release 15. College Station, TX: StataCorp LLC).

### Ethical approval and consent to participate

The research was performed in accordance with the Utah University research ethic guideline. Research have been performed in accordance with the Declaration of Helsinki. This study was approved by the Institutional Review Board of Utah university. Informed consent was waived for all participants and approved by the ethic committee of the Utah University Institutional Review Board (number IRB_00137807).

## Results

From October 1999 to December 2020, a total of 566 patients with AF who were concurrently diagnosed with active cancer were included in this study. The median follow-up duration was 48.9 months (interquartile range 22.2–73.9 months). A summary of the clinical characteristics of the patients is presented in Table 1. The mean age of the participants was 76.2 years, with a standard deviation (SD) of 10.7. Among the included patients, 326 were males, accounting for 57.6% of the cohort. The mean CHA_2_DS_2_-VASc Score was 4.4 (SD 2.0). It was observed that patients who received LMWH/UFH at the time of cancer diagnosis were slightly younger and had a higher likelihood of having chronic kidney disease compared to those who were treated with warfarin or DOAC.

**Table 1:** Clinical characteristics of patients.

Clinical characters	Total (n = 566)	Warfarin (n = 219)	DOAC (n = 106)	UFH/LMWH (n = 241)
Age				
Mean (SD)	76.2 (10.7)	79.4 (10.2)	76.1 (8.8)	73.5 (11.3)
Median (IQR)	77.0 (69.0–83.0)	80.0 (74.0–87.0)	77.0 (70.0–82.0)	74.0 (66.0–80.0)
Male, n (%)	326 (57.6)	122 (55.7)	63 (59.4)	141 (58.5)
Medical history, n (%)				
Diabetes	65 (11.5)	15 (6.9)	16 (15.1)	34 (14.1)
Hypertension	414 (73.1)	167 (76.3)	78 (73.6)	169 (70.1)
Dyslipidemia	84 (14.8)	17 (7.8)	28 (26.4)	39 (16.2)
Ischemic heart disease	219 (38.7)	95 (43.4)	37 (34.9)	87 (36.1)
Stroke	75 (13.3)	32 (14.6)	16 (15.1)	27 (11.2)
Venous thrombosis	189 (33.4)	87 (39.7)	28 (26.4)	74 (30.7)
PAD	151 (26.7)	58 (26.5)	27 (25.5)	66 (27.4)
Chronic kidney disease	79 (13.9)	23 (10.5)	11 (10.4)	45 (18.7)
CHA_2_DS_2_-VASc score				
Mean (SD)	4.4 (2.0)	4.6 (1.9)	3.9 (2.2)	4.4 (2.0)
0	9 (1.6)	1 (0.9)	1 (0.9)	7 (2.9)
1	38 (6.7)	6 (2.8)	16 (15.1)	16 (6.7)
2	62 (11.0)	27 (12.4)	17 (16.0)	18 (7.5)
3	84 (14.9)	34 (15.6)	17 (16.0)	33 (13.8)
4	110 (19.5)	45 (20.6)	16 (15.1)	49 (20.4)
5	96 (17.0)	37 (17.0)	12 (11.3)	47 (19.6)
6	73 (12.9)	26 (11.9)	11 (10.4)	36 (15.0)
7	58 (10.3)	29 (13.3)	8 (7.6)	21 (8.8)
8	25 (4.4)	10 (4.6)	7 (6.6)	8 (3.3)
9	9 (1.6)	3 (1.4)	1 (0.9)	5 (2.1)
Primary cancer site				
Melanoma	16 (2.8)	6 (2.7)	2 (1.9)	8 (3.3)
Breast	37 (6.5)	14 (6.4)	5 (4.7)	18 (7.5)
Colon	13 (2.3)	3 (1.4)	0	10 (4.2)
Gynecology	30 (5.3)	6 (2.7)	6 (5.7)	18 (7.5)
Prostate	46 (8.1)	23 (10.5)	10 (9.4)	13 (5.4)
Male genital	1 (0.2)	0	0	1 (0.4)
Head and neck	15 (2.7)	5 (2.3)	3 (2.8)	7 (2.9)
Urinary tract	26 (4.6)	5 (2.3)	7 (6.6)	14 (5.8)
Non-colonic gastrointestinal	34 (6.0)	15 (6.9)	3 (2.8)	16 (6.6)
Brain	10 (1.8)	2 (0.9)	1 (0.9)	7 (2.9)
Bone	46 (8.1)	32 (14.6)	3 (2.8)	11 (4.6)
Endocrine	13 (2.3)	3 (1.4)	2 (1.9)	8 (3.3)
Pleura	1 (0.2)	0	0	1 (0.4)
Non specific	40 (7.1)	19 (8.7)	7 (6.6)	14 (5.8)
Lymphatic spreading	14 (2.5)	1 (0.5)	4 (3.8)	9 (3.7)
Secondary cancer	15 (2.7)	4 (1.8)	3 (2.8)	8 (3.3)
Lymphoma	52 (9.2)	13 (5.9)	12 (11.3)	27 (11.2)
Others	157 (27.7)	68 (31.1)	38 (35.8)	51 (21.2)

*DOAC* direct oral anticoagulant, *UFH* unfractionated heparin, *LMWH* low-molecular-weight heparin, *SD* standard deviation, *IQR* interquartile range, *PAD* peripheral arterial disease.

### Pattern of anticoagulant prescription

**Figure 1:**
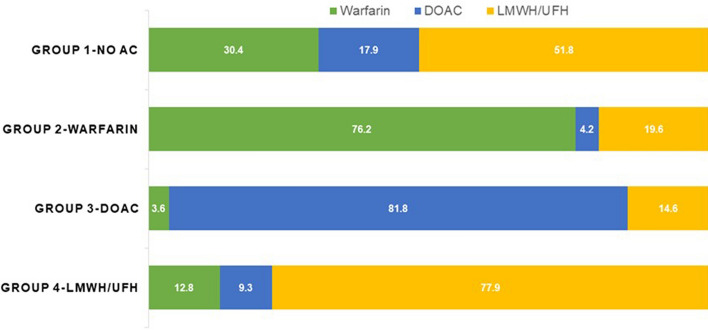
Pattern of anticoagulant use in cancer patients with atrial fibrillation. Group 1 patients who did not received anticoagulant, group 2 patients who received warfarin before cancer diagnosis, group 3 patients who received DOACs before cancer diagnosis and group 4 patients who received LMWH/UFH before cancer diagnosis. *NO AC* no anticoagulant, *DOAC* direct oral anticoagulant, *LMWH* low-molecular-weight heparin, *UFH* unfractionated heparin.

Figure 1 summarizes anticoagulant prescribing patterns in AF patients who concurrently had active cancer.

Among patients who did not receive anticoagulant before index date (Group 1, 257 patients), 30.4% received warfarin, 17.9% received DOACs and 51.8% received LMWH/UFH.

Among patients who received warfarin before index date (Group 2, 168 patients), 76.2% continued to receive warfarin, 4.2% received DOACs and 19.6% received LMWH/UFH.

Among patients who received DOACs before index date (Group 3, 55 patients), 3.6% switched to warfarin, 81.8% continued to receive DOACs and 14.6% received LMWH/UFH.

Among patients who received LMWH/UFH (Group 4, 86 patients), 12.8% switched to warfarin, 9.3% switched to DOACs and 77.9% continued to receive LMWH/UFH.

### Stroke

Univariable Cox-proportional hazards analysis identified type of anticoagulant, higher CHA_2_DS_2_-VASc Score, chronic kidney disease, and dyslipidemia were independently associated with higher stroke risk. The HR of stroke associated with CHA_2_DS_2_-VASc score was 1.3 (95% CI 1.1–1.4, P = 0.001) meaning that each 1-point increasing in the CHA_2_DS_2_-VASc score, was associated with a 30.0% stroke risk increase. HRs were 2.5 (95% CI 1.3–4.8, P = 0.006) for chronic kidney disease and 2.5 (95% CI 1.1–5.5, P = 0.021) for dyslipidemia. After adjusting for co-variates (CHA_2_DS_2_-VASc score, chronic kidney disease and dyslipidemia) in the MSM analysis, LMWH/UFH use was associated with significantly higher risk of stroke, adjusted HR (aHR) 2.4 (95% CI, 1.0–5.6, P = 0.04) compared to warfarin. The use of DOACs was not associate with an increase risk of stroke as compared to warfarin with aHR of 0.8 (95% CI 0.2–2.7, P = 0.67).

### Gastrointestinal (GI) bleeding

The rate of GI bleeding was 3.8, 5.5 and 8.3 events per 100 patient-years in patients who received warfarin, DOACs and heparin, respectively (Table 2). Univariable analysis revealed primary GI cancer was associated with higher risk of GI bleeding (HR 2.1, 95% CI 1.2–3.7, P = 0.009).

**Table 2:** Clinical outcomes in atrial fibrillation patients who had active cancer.

	Patient-years	Number of event	Event rate (event/100 PY)	Unadjusted HR (95% CI)	aHR-MSM (95% CI)
Stroke					
Warfarin	1353	19	1.4 (0.9–2.2)	Reference	Reference
DOAC	471	9	2.7 (1.0–3.7)	1.3 (0.6–3.0)	0.8 (0.2–2.7)
LMWH/UFH	319	16	5.0 (3.1–8.2)	3.1 (1.6–6.0)	2.4 (1.0–5.6)
GI-bleeding					
Warfarin	1217	47	3.8 (2.9–5.1)	Reference	Reference
DOAC	434	24	5.5 (3.7–8.2)	1.4 (0.8–2.3)	1.5 (0.9–2.6)
LMWH/UFH	276	23	8.3 (5.5–12.5)	1.9 (1.2–3.2)	1.2 (0.6–2.4)
Intracranial bleeding					
Warfarin	1370	22	1.6 (1.0–2.4)	Reference	Reference
DOAC	492	6	1.2 (0.5–2.7)	0.7 (0.3–1.8)	0.8 (0.3–2.5)
LMWH/UFH	333	10	3.0 (1.6–5.6)	1.7 (0.8–3.5)	1.1 (0.4–3.1)
Overall -bleeding					
Warfarin	973	112	11.5 (9.5–13.8)	Reference	Reference
DOAC	316	45	14.2 (10.6–19.0)	1.2 (0.8–1.7)	1.1 (0.7–1.6)
LMWH/UFH	197	49	24.8 (18.8–32.9)	2.0 (1.4–2.8)	1.1 (0.6–1.7)
Death					
Warfarin	1419	57	4.0 (3.1–5.2)	Reference	Reference
DOAC	516	28	5.4 (3.7–7.8)	1.4 (0.9–2.2)	1.2 (0.7–2.2)
LMWH/UFH	349	50	14.3 (10.8–18.9)	4.1 (2.7–6.1)	4.5 (2.8–7.2)

*GI* gastrointestinal, *DOAC* direct oral anticoagulant, *LMWH* low-molecular-weight heparin, *UFH* unfractionated heparin, *PY* patient-years, *HR* hazard ratio, *aHR* adjusted subdistribution hazard ratio, *CI* confidence interval.

Multivariable, MSM analysis showed that DOACs and heparin did not significantly increase risk of GI bleeding as compared to warfarin, aHR 1.5 (95% CI 0.9–2.6, P = 0.14 and aHR 1.2 (95% CI 0.6–2.4, P = 0.56), respectively.

### Intracranial bleeding

The rate of intracranial bleeding was 1.6, 1.2 and 3.0 events per 100 patient-years in patients who received warfarin, DOACs and heparin, respectively (Table 2). Univariable analysis revealed CKD and primary brain cancer were associated with higher intracranial bleeding risk (HRs 2.4 95% CI 1.2–5.0, P = 0.013 and 4.9 95% CI 1.2–20.5, P = 0.027, respectively).

Multivariable, competing-risk analysis showed that DOACs and LMWH/UFH did not significantly increase risk of CNS bleeding as compared to warfarin, aHR 0.8 (95% CI 0.3–2.5, P = 0.73) and aHR 1.1 (95% CI 0.4–3.1, P = 0.76), respectively.

### Overall bleeding

Overall bleeding occurred at rates of 11.5, 14.2 and 24.8 events per 100 patient-years in patients who received warfarin, DOACs and LMWH/UFH, respectively (Table 2). Univariable analysis revealed that primary GI cancer was associated with increased risk of overall bleeding, HR 2.0 (95% CI 1.3–3.0, P = 0.02).

Multivariable, competing-risk analysis showed that DOACs and LMWH/UFH did not significantly increase risk of overall bleeding as compared to warfarin, aHR 1.1 (95% CI 0.7–1.6, P = 0.73) and aHR 1.1 (95% CI 0.6–1.7, P = 0.83), respectively.

### Mortality

All-cause mortality occurred at rates of 4.0, 5.4 and 14.3 events per 100 patient-years in patients who received warfarin, DOACs and LMWH/UFH, respectively (Table 2). Univariable analysis revealed higher CHA_2_DS_2_-VASc Score and chronic kidney disease were associated with increased risk of death, HR 1.2 (95% CI 1.1–1.3, P < 0.001) and HR 2.3 (95% CI 1.6–3.5, P < 0.001), respectively.

Multivariable, competing-risk analysis showed that LMWH/UFH use was associated with an increased risk of death as compared to warfarin, aHR 4.5 (95% CI 2.8–7.2, P < 0.001) but not for DOACs (aHR 1.2 95% CI 0.7–2.2, P = 0.47).

We conducted a sensitivity analysis by excluding participants who were enrolled before 2010. The results of this analysis remained consistent with the findings of the primary analysis for all outcomes. (data not shown).

## Discussion

The optimal anticoagulant strategy in patients with cancer who also have AF remains controversial. This study demonstrates that anticoagulants might be switched after cancer diagnosis. We observed an increased risk for stroke and all-cause mortality in patients using LMWH/UFH compared to warfarin.

Previously, a retrospective study of 472 cancer patients with electrocardiography-documented AF or atrial flutter (AFL) revealed that 44.3% of patients did not receive stroke prevention with anticoagulant therapy^12^. Likewise, in a prospective study of 4664 cancer patients, 394 of which had documented AF^13^, only 40% of AF patients received anticoagulant. These investigators found that anticoagulant treatment was not significantly related to mortality^13^. Possible reasons for this concerning lack of prescribing anticoagulant therapy for more than half of cancer patients who had AF may have included current chemotherapy use, prior bleeding, and co-morbid conditions^12^.

Our observations indicated that patients who initiated anticoagulant therapy after their cancer diagnosis were more likely to receive LMWH/UFH compared to warfarin or DOACs. Conversely, individuals who were already on anticoagulant treatment before their cancer diagnosis were more inclined to continue with the same anticoagulant regimen.

Patients with cancer may be more likely to be prescribed LMWH because it is preferred for treatment of VTE in this patient population^14^. However, evidence supporting the use of LMWH as a prophylaxis for stroke prevention in any patient with AF is scanty. Our main finding shows that patients who were prescribed LMWH or UFH (LMWH/UFH group) had a fourfold increased risk of stroke as compared to warfarin. The use of DOACs did not significantly increase risk of stroke compared to warfarin.

This finding was consistent with results from a prospective multicenter registry that included a total of 320 patients with active cancer and also had AF. Of these 192 patients were treated with DOACs and 110 patients with LMWH^15^. Stroke or systemic embolism occurred 1.0% per year in the DOACs group and 7.2% per year in LMWH group (P < 0.05). The bleeding rate was not statistically different between two groups^15^. This study emphasized that the use of LMWH might not be optimal for the prevention of stroke in cancer patient with AF.

The evidence supporting DOACs for stroke prevention in patients with cancer and AF is not strong because most patients with cancer were excluded from pivotal studies^16^. Post-hoc analysis of data from ENGAGE AF-TIMI 38 study included 1153 patients with AF who had new or recurrent malignancy^17^. The annualized stroke and systemic embolism rate was 1.4% per year in patients who received edoxaban 60 mg once daily, 2.0% per year in patients who received edoxaban 30 mg once daily, and 2.4% per year in those who received warfarin^17^. There was no statistical difference in efficacy between both doses of edoxaban and warfarin^17^. Another study analyzing 1236 patients with AF who had cancer demonstrated that there was no difference in the rate of stroke and systemic embolism in patients treated with apixaban as compared to warfarin (1.4% vs 1.2% per year, HR 1.1, 95% CI 0.5–2.3)^18^.

Our study found that patients with cancer who also had AF and received any type of anticoagulant had an increased risk of bleeding. However, there was no statistical difference between bleeding rates in patients receiving either DOACs or LMWH/UFH as compared to warfarin. These findings were consistent with post-hoc analyses of the ENGAGE AF-TIMI 38 and ARISTOTLE trials which reported comparable bleeding rates between cancer patients who received DOACs (edoxaban or apixaban) vs. warfarin^17,18^. We also found that patients with primary GI cancer and CNS cancer were more likely to have GI and CNS bleeding, respectively. Data from pivotal trials in patients with cancer associated venous thrombosis showed that patients using edoxaban and rivaroxaban but not apixaban had increased risk of major bleeding as compared to LMWH, especially patients with GI or GU cancer^19–21^. However, an analysis of a medical record database in Taiwan demonstrated that the use of DOACs was associated with similar risk for major bleeding compared with LMWH^22^. The findings from real-world evidence may better reflect the appropriateness of anticoagulant choice in individual patients with different underlying bleeding risks.

The risk of death from any cause in our study was fourfold higher in the group prescribed LMWH/UFH compared to those prescribed warfarin. The death rate for warfarin and DOACs observed in our study was comparable from a post-hoc analysis of the ARISTOTLE trial in patients with AF who had cancer, which reported 4.7 and 3.6 deaths per 100 patient-years in patients who received apixaban and warfarin, respectively^18^. The reasons underlying the higher death rate associated with LMWH/UFH in our study were unclear but might be explained from patients’ underlying conditions or anticoagulant effectiveness.

One of the strengths of this study is that it provided real-world data on the usage of anticoagulants among cancer patients who also have atrial fibrillation, both prior to and following a cancer diagnosis.

Our study has some limitations. First, we did not perform subgroup analysis by the four different types of DOACs because of the small numbers of patients in each category. Second, we did not include cancer patients with AF who did not receive anticoagulant therapy. Therefore, we could not compare the risk of stroke/systemic embolism and bleeding between these two groups. Third, the use of anticoagulant was based on prescription records. Therefore, we could not estimate the compliance rate and impact of compliance on outcomes. Fourth, given the retrospective nature of our data collection, we encountered limitations in obtaining comprehensive data required to classify the severity of bleeding according to the International Society on Thrombosis and Haemostasis (ISTH) guideline^23^. However, it is important to note that the bleeding outcomes we captured were of sufficient severity to necessitate hospitalization, which we considered clinically relevant. Finally, the participants were enrolled between 1999 and 2020. Considering that DOACs were approved for the prevention of stroke and systemic embolism in 2010, patients enrolled during that period were more likely to receive warfarin treatment. However, it is important to note that the proportion of patients from that specific period was relatively small. Moreover, the sensitivity analysis conducted after excluding those patients yielded results consistent with the primary analysis.

## Conclusions

This study found that LMWH/UFH, compared to warfarin in cancer patients with AF, was associated with increased risk of stroke. Furthermore, DOACs was associated with similar risk of stroke and bleeding as compared to warfarin. Based on our findings, we recommend caution in the use of LMWH/UFH in cancer patients with AF. Further prospective trials are needed to confirm these findings.

## Data Availability

The datasets generated and/or analysed during the current study are not publicly available due to institutional restriction but are available from the corresponding author on reasonable request.
